# Long-Chain Metabolites of Vitamin E: Metabolic Activation as a General Concept for Lipid-Soluble Vitamins?

**DOI:** 10.3390/antiox7010010

**Published:** 2018-01-12

**Authors:** Martin Schubert, Stefan Kluge, Lisa Schmölz, Maria Wallert, Francesco Galli, Marc Birringer, Stefan Lorkowski

**Affiliations:** 1 Department of Biochemistry and Physiology of Nutrition, Friedrich-Schiller-University Jena, 07743 Jena, Germany; m.schubert@uni-jena.de (M.S.); s.kluge@uni-jena.de (S.K.); lisa.schmoelz@uni-jena.de (L.S.); maria.wallert@uni-jena.de (M.W.); 2 Competence Center for Nutrition and Cardiovascular Health (nutriCARD), Halle-Jena-Leipzig, 07743 Jena, Germany; 3 Baker IDI Heart and Diabetes Institute, Melbourne VIC 3004, Australia; 4 Department of Pharmaceutical Sciences, Laboratory of Nutrition and Clinical Biochemistry, University of Perugia, 06123 Perugia, Italy; francesco.galli@unipg.it; 5 Department of Nutrition, Food and Consumer Sciences, University of Applied Sciences Fulda, 36037 Fulda, Germany; marc.birringer@oe.hs-fulda.de

**Keywords:** vitamin E, long-chain metabolites of vitamin E, 13′-hydroxychromanol (13′-OH), 13′-carboxychromanol (13′-COOH), vitamin E metabolism, biological activity

## Abstract

Vitamins E, A, D and K comprise the class of lipid-soluble vitamins. For vitamins A and D, a metabolic conversion of precursors to active metabolites has already been described. During the metabolism of vitamin E, the long-chain metabolites (LCMs) 13′-hydroxychromanol (13′-OH) and 13′-carboxychromanol (13′-COOH) are formed by oxidative modification of the side-chain. The occurrence of these metabolites in human serum indicates a physiological relevance. Indeed, effects of the LCMs on lipid metabolism, apoptosis, proliferation and inflammatory actions as well as tocopherol and xenobiotic metabolism have been shown. Interestingly, there are several parallels between the actions of the LCMs of vitamin E and the active metabolites of vitamin A and D. The recent findings that the LCMs exert effects different from that of their precursors support their putative role as regulatory metabolites. Hence, it could be proposed that the mode of action of the LCMs might be mediated by a mechanism similar to vitamin A and D metabolites. If the physiological relevance and this concept of action of the LCMs can be confirmed, a general concept of activation of lipid-soluble vitamins via their metabolites might be deduced.

## 1. The Biological Significance of Vitamin E

The term vitamin E comprises eight lipophilic molecules, which can be classified as tocopherols (TOHs) and tocotrienols (T3). Both classes share two common features: (i) the phytyl-like side chain, which is bound to (ii) the chroman ring system. A saturated side chain characterizes the TOHs, while the T3s carry three double bonds in this substructure. Further, the methylation pattern of the chroman ring determines the classification as α-, β-, γ- or δ-TOH or T3, respectively. Vitamin E is found in oils, nuts, germs, seeds and a variety of other plant products. The naturally found vitamin E forms exist either in *RRR*-configuration (TOHs) or in *R*-configuration (T3s), whereas only synthetically produced forms contain a mixture of the different possible stereoisomers [1]. 

Vitamin E was discovered in 1922 as vital factor for the fertility of rats, indicating its essentiality for animal and human health, and was therefore classified as a vitamin [2]. Nevertheless, the benefits of vitamin E for human health are still a contentious issue. However, several disease conditions, such as anemia, erythrocyte rupture and neuronal degeneration, as well as muscle degeneration, are linked to vitamin E deficiency or malabsorption (extensively reviewed in [3]). Further, vitamin E was shown in human intervention trials to slow down the progression of age-related neurodegenerative pathologies such as Alzheimer’s disease, maybe due to its antioxidative properties [4,5]. Vitamin E is also an essential factor for the development of the central nervous system and cognitive functions of the embryo [6,7]. Next, vitamin E may play a supportive role in the prevention of neural tube defects in humans along with folic acid [8,9]. Initially, the effects of vitamin E were only attributed to its antioxidant properties, however more recent work unveiled non-antioxidant regulatory effects. There is growing evidence that vitamin E modulates gene expression and enzyme activities and interferes with signaling cascades independent of its capacity as an antioxidant [10]. Over time, several functions of vitamin E, such as suppression of inflammatory mediators, reactive oxygen species, and adhesion molecules, the induction of scavenger receptors, and the activation of nuclear factor kappa-light-chain-enhancer of activated B cells (NF_k_B) (reviewed in [11]) were revealed. Based on these observations, it was concluded that vitamin E likely plays a role in several inflammatory but also other diseases. However, further research is required, as the results obtained from clinical trials with TOHs are inconsistent with respect to beneficial effects on the development of chronic diseases such as cancer and cardiovascular diseases [12].

## 2. Absorption and Distribution of Vitamin E

Like for all macro- and micronutrients, intestinal absorption is the limiting factor for the bioavailability of vitamin E in humans. As a fat-soluble vitamin, intestinal absorption, hepatic metabolism and cellular uptake of vitamin E follows that of other lipophilic molecules [13]. The absorption rate of vitamin E varies between 20% and 80% [13,14], and is thus generally lower than for vitamins A and D [15,16]. Differences in the rates of absorption of vitamin E and the other fat-soluble vitamins may result also from the parallel intake of additional food ingredients. For example, retinoic acid [17], plant sterols [18], eicosapentaenoic acid [14], alcohol (chronic consumption) [14], and dietary fiber [19] are natural food components that may compete with the absorption of vitamin E. In addition, it has been shown that the supplied form of vitamin E, either as a free molecule or coupled to other compounds like acetate, is also crucial for its bioavailability [20]. 

For optimal absorption, fat must be consumed along with the ingested vitamin E. This is a general requirement for all types of fat-soluble vitamins and is therefore also applicable for vitamins A, D and K [16,21]. The absorption of triacylglycerides and esterified fat-soluble molecules starts with enzymatic processing in the stomach by the action of gastric lipases [15]. The following digestion of dietary lipids appears in the intestinal lumen by the action of various enzymes, including pancreatic lipase, carboxyl esterase and phospholipase A_2_ [22]. Since most of the vitamin E in the human diet is not esterified, lipolytic degradation is scarce [14]. In contrast, the human diet contains significantly more esterified vitamin A and D, mostly in the form of retinyl-esters and vitamin D_3_ oleate, which can be hydrolyzed by the above mentioned enzymes [16,21]. A key step of the intestinal absorption of fat-soluble vitamins is the emulsification, i.e., the incorporation into micelles formed with phospholipids and bile acids. Under normal conditions, bile salts facilitate the absorption of all three vitamins, but especially the vitamin D forms differ in their dependency for bile salt availability, i.e., vitamin D_3_ absorption is more dependent on the presence of bile salts than 25-hydroxyvitamin D (OHD) [23]. After emulsification, vitamin E is taken up into the intestinal enterocytes by passive diffusion or receptor-mediated transport via scavenger receptor class B type 1 (SRB1) [24], or Niemann–Pick C1-like protein 1 [25], which is also involved in the uptake of the vitamins A, D and K as well as cholesterol [16,26,27]. Since no specific plasma transport protein for α-TOH is known, the subsequent transport of vitamin E in blood follows largely that of cholesterol [25], meaning that under normal physiological conditions, α-TOH is transported via chylomicrons. This transport is independent of the type of stereoisomer [28,29]. In addition, retinol, unconverted pro-retinoid carotenoids (β-carotene), non-pro-retinoid carotenoids (lycopene), vitamin D_3_ and phylloquinone (representing the main dietary form of vitamin K) are also incorporated into chylomicrons [16,21,30]. After entering the circulation, chylomicrons undergo a process of remodeling that involves primarily the hydrolysis of triglycerides by lipoprotein lipase, resulting in the formation of chylomicron remnants [25]. Vitamins E, A, D and K are not affected by hydrolysis and remain in the lipoprotein particle for further transport to the liver [31]. The different forms of vitamin E are discriminated in the liver by the α-tocopherol transfer protein (α-TTP), which promotes the incorporation of 2*R*- or *RRR*-α-TOH into very low-density lipoproteins (VLDL) [32,33], whereas other forms and stereoisomers are either metabolized or secreted into bile [34]. Besides α-TTP, the TOH-associated protein and the TOH-binding protein are known mediators of the intracellular transport of vitamin E. Interestingly, α-TOH secretion from the liver is apparently not necessarily dependent on VLDL assembly and secretion, thus oxysterol-binding proteins [35] and ATP-binding cassette transporter A1 (ABCA1) [36] have been suggested to contribute to the release from the liver. Furthermore, ABCA1 mediates the efflux of vitamin E in the intestine, macrophages, and fibroblasts [36], and multidrug resistance P-glycoprotein has been identified as a transporter for the excretion of α-TOH via bile [37]. After the release of vitamin E-carrying VLDL into blood circulation and action of lipoprotein lipase as well as hepatic lipase, receptors such as SRB1, low-density lipoprotein (LDL) receptor as well as LDL receptor-related protein mediate the uptake of vitamin E into peripheral tissues and the liver [31,38]. 

## 3. Metabolism of Vitamin E

**Figure 1:**
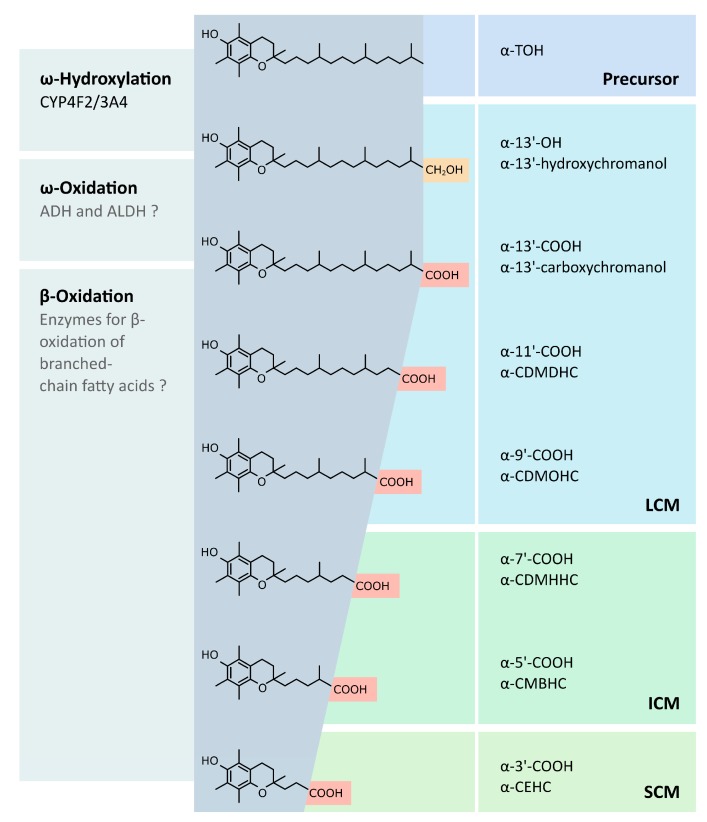
Metabolism of vitamin E. The metabolism of vitamin E is initiated by a terminal ω-hydroxylation of the side-chain via CYP4F2 and CYP3A4. The resulting hydroxychromanol is further modified by ω-oxidation, resulting in the formation of carboxychromanol, possibly by alcohol and aldehyde dehydrogenases. As a consequence, the metabolite can be subjected to β-oxidation. Five cycles of β-oxidation lead to the formation of the short-chain metabolite CEHC. However, this review focuses on the LCMs 13′-OH and 13′-COOH as these molecules have been synthesized in sufficient amounts for in vitro and in vivo investigations. The following abbreviations are used: ADH, alcohol dehydrogenase; ALDH, aldehyde dehydrogenase; CDMDHC, carboxydimethyldecylhydroxychromanol; CDMOHC, carboxymethyloctylhydroxychromanol; CDMHHC, carboxymethylhexylhydroxychromanol; CMBHC, carboxymethylbutylhydroxychromanol; CEHC, carboxyethylhydroxychromanol.

The metabolism of vitamin E is primarily localized in the liver (Figure 1) (reviewed in [39]), whereas extrahepatic pathways have been also suggested [40,41]. The degradation processes of hepatic metabolism remain poorly understood, but the initial mechanisms are generally accepted, i.e., all vitamers are degraded to vitamer-specific physiological metabolites with an intact chromanol ring and a shortened side-chain. Interestingly, accumulation of vitamin E to toxic levels is prevented by increased metabolism in response to higher vitamin E levels. Due to the preferential binding to α-TTP, α-TOH is the prevalent form of vitamin E in humans. It is speculated that α-TTP protects the α-form from degradation, thus leading to the accumulation of α-TOH. With the lower affinities of the other vitamin E forms to α-TTP taken into consideration, γ- and δ-forms are likely catabolized faster [42]. Despite of the different catabolic rates, all forms of vitamin E follow the same metabolic route, as confirmed by the detection of the respective end products of hepatic metabolism, α-, β-, γ-, and δ-carboxyethylhydroxychromanol (CEHC) [43,44]. However, the rate of catabolism is different for the vitamin E forms, possibly due to distinct affinities to key enzymes [42,45]. The chroman ring is not modified during catabolism (the catabolic end products are still classified as α-, β-, γ- and δ-forms); it is rather the aliphatic side chain where modifications are introduced. Metabolism of T3 follows the same principle, albeit further enzymes such as 2,4 dienoyl-coenzyme A (CoA) reductase and 3,2-enoyl-CoA isomerase (necessary for the metabolism of unsaturated fatty acids) are likely required for the degradation of the unsaturated side chain [46]. 

The catabolism of the vitamin E molecule takes place in different cell compartments: endoplasmic reticulum, peroxisomes, and mitochondria. However, the mechanism of metabolite transfer between the compartments is not well understood and requires further investigation. The initial step at the endoplasmic reticulum leads to the formation of 13′-hydroxychromanol (13′-OH) metabolites via ω-hydroxylation by cytochrome P450 (CYP) 4F2 or CYP3A4, respectively [45,47]. The following ω-oxidation, which is probably mediated by alcohol and aldehyde dehydrogenases (an aldehyde intermediate is formed), results in 13′-carboxychromanol (COOH) metabolites. In general, the resulting metabolites with carboxy function are degraded like branched-chain fatty acids. Hence, the side chain is shortened by β-oxidation, and the formed propionyl-CoA or acetyl-CoA is eliminated. The intermediate-chain metabolites 11′-COOH and 9′-COOH are formed in peroxisomes during the first two cycles of β-oxidation. Three additional cycles of β-oxidation are carried out in the mitochondria, resulting in the short-chain metabolites (SCMs) 7′-COOH and 5′-COOH as well as the end-product CEHC or 3′-COOH. Moreover, conjugation of the metabolites takes place during metabolism, resulting predominantly in sulfated and glucuronidated metabolites. However, glycine-, glycine–glucuronide-, and taurine-modified metabolites of vitamin E have also been identified [48].

The conjugated SCMs are more hydrophilic and thus mainly found in glucuronidated form in human urine [44]. In contrast, the long-chain metabolites (LCMs) and their metabolic precursors are secreted via bile into the intestine and the metabolites in fecal samples are not conjugated. The fecal route is considered as the major pathway of vitamin E excretion [12,49].

Like vitamin E, other fat-soluble vitamins, such as the vitamins A (i), D (ii) and K (iii) are also metabolized in the human body:

(i). Under physiological conditions, retinyl esters (in the intestinal lumen) and carotenoids (in enterocytes) are converted into retinol before or during their intestinal absorption, respectively. Inside the enterocytes, retinol is re-esterified by lecithin-retinol acyl transferase or acyl-CoA:retinol-acyltransferase and packed into chylomicrons for transport. The retinyl esters are transferred to the liver and stored in hepatic parenchymal and non-parenchymal cells. Vitamin A is mobilized from liver stores by the retinol-binding protein, a specific transporter allowing the transport of retinol in blood circulation [50]. These results suggest that vitamin A has an active (retinol) and a storage form (retinyl ester). In addition, the oxidation of retinol leads to the formation of retinal, another active form of vitamin A, which is primarily bound to opsins in the photoreceptors of the retina [51]. More current research indicates that all-*trans* retinoic acid (ATRA), 9-*cis*-RA, and all-*trans*-4-oxo-RA are the vitamin A metabolites with the highest biological activity. These active vitamin A metabolites serve as ligands for nuclear receptors, called retinoic acid receptors (RARs) [52] and retinoid receptors (RXRs) [53], which act as ligand-activated transcription factors controlling the expression of their respective target genes. Therefore, hepatic retinol is transferred to extrahepatic tissues and metabolized to retinoic acid by different enzymatic systems. Lampen and co-workers found that ATRA is also formed in the small intestine via direct oxidation of vitamin A. Based on this result, they hypothesized that biologically active retinoids are formed in the gastrointestinal tract and act as retinoid-receptor ligands controlling various processes in the intestinal mucosa via RAR [53].
(ii). The human metabolism of vitamin D is primarily located in liver and kidney. Metabolism of vitamin D_2_ and D_3_ starts with the formation of 25-OHD, the major circulating vitamin D metabolite, by vitamin D-25 hydroxylase. Afterwards, 25-OHD is transferred to the kidney and further catabolized by 25-OHD-1α-hydroxylase to 1,25-dihydroxyvitamin D_2/3_. These molecules serve as ligands for the vitamin D receptor (VDR), a transcription factor expressed in various tissues. Vitamin D receptor binds to specific regions in the promoter regions of genes, the so-called vitamin D responsive elements, thus controlling the expression of respective target genes. Therefore, 1,25-dihydroxyvitamin D is the active metabolic form of vitamin D [54,55].
(iii). Phylloquinone (vitamin K_1_) and menaquinone (vitamin K_2_) are summarized by the term vitamin K. Phylloquinone is synthesized in plants, while menaquinone is derived from animal and bacterial origins [30,56]. Both compounds share a 2-methyl-1,4-naphthoquinone structure, called menadione, and a side chain at the 3′-position. The side chain of phylloquinone is composed of three isopentyl units and one isopentenyl unit, while the side chain of menaquinone contains a variable number of only isopentenyl units (2–13) [30]. The metabolism of vitamin K is localized in the liver and has not been studied in detail so far [57]. Nevertheless, the metabolic pathway of phylloquinone and menaquinone degradation likely follows that of vitamin E. Hence, the degradation starts with an initial ω-oxidation, which is mediated by CYP. While the ω-oxidation of vitamin E is catalyzed primarily by CYP4F2, CYP3A4 has been described as the possible mediator for the ω-oxidation of vitamin K. Next, the following degradation of the side chain of vitamin K occurs via β-oxidation [30,56,58]. A 5-carbon carboxylic acid metabolite termed K acid 2 has been identified as the end-product of either phylloquinone or menaquinone metabolism and is excreted via urine and bile [30,58]. In addition to their metabolic degradation, it has been suggested that phylloquinones could also be converted to menaquinones [59,60]. For this, phylloquinone is likely transformed to the intermediate menadione by removing its side chain, which is subsequently replaced by a newly synthesized isopentenyl side chain to form menaquinone [30]. While menaquinone is considered as the physiologically active form of vitamin K in humans [56], almost nothing is known about a possible biological activity of the vitamin K metabolites. Further studies are needed to unravel whether vitamin K must be included into the general concept of a metabolic pre-activation of lipid-soluble vitamins.

Although the metabolisms of vitamin A and D differ in location and the involved enzymatic systems, the formation of active metabolites seems to be a key element of both metabolic pathways, i.e., both vitamins mediate their gene regulatory effects by metabolic pre-activation. Therefore, the discovery of vitamin E metabolism in animals and humans and the emerging evidence for important biological functions of vitamin E metabolites could indicate a general metabolic activation mechanism of fat-soluble vitamins in the human body.

### In Vivo Verification of Systemic LCM Availability

Since the discovery of vitamin E by Evans and Bishop in 1922 [2], α-TOH has been accounted as an antioxidant capable to scavenge reactive oxygen species, and decreased α-TOH levels have been associated with several diseases including different types of cancer, cardiovascular diseases and diabetes [61]. It took 80 years since Azzi and co-workers set up the hypothesis for an additional gene regulatory role of α-TOH in the human body [62]. In addition, the discovery of vitamin E metabolism in animals and humans and the emerging evidence for important biological functions of the vitamin E metabolites [63,64], suggested that the TOHs may gain biological activity after metabolism (as confirmed for vitamin A and D). This prompted studies that investigated also the putative functions of the LCMs of TOH. In 2014, Wallert and co-workers showed the occurrence of α-13′-COOH in human serum, which has been confirmed later by others [65,66]. For these studies, serum obtained from a healthy, middle-aged (39 years), non-smoking male, who received a balanced diet with no additional vitamin E supplementation was used for the detection of α-13′-COOH via liquid chromatography coupled mass spectrometry [63]. The analyses revealed for the first time that α-TOH metabolites are transferred into blood circulation following metabolism of α-TOH in the liver. Furthermore, cell experiments showed that α-13′-OH and α-13′-COOH are more potent regulators of gene expression than their metabolic precursor α-TOH [63]. Taken together, the results of Wallert et al. provided the first evidence that the LCMs are an active form of their metabolic precursor [63], promoting regulatory effects in peripheral tissues of the human body. However, while the role of vitamin E as a lipophilic antioxidant in vitro is widely accepted, the relevance in vivo is still a matter of debate [67,68,69].

## 4. Biological Activity

**Figure 2:**
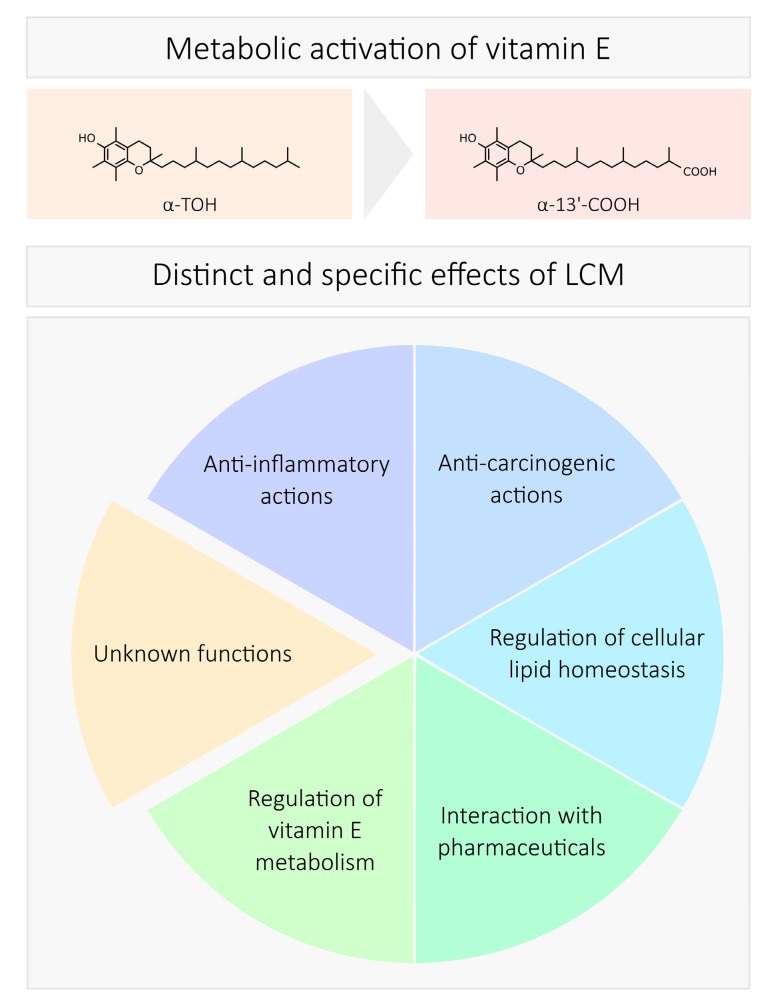
Reported biological functions of the LCMs of vitamin E.

Not much is known about the biological activity of the LCMs. However, the publications on this topic published during the last ten years can be categorized by the biological effects of the LCMs as follows: (i) anti-inflammatory actions [64,70,71,72,73,74,75]; (ii) anti-carcinogenic effects [72,76,77]; (iii) regulation of cellular lipid homeostasis [63,64]; (iv) interaction with pharmaceuticals [78]; and (v) regulation of their own metabolism [79] (Figure 2). 

### 4.1. Anti-Inflammatory Actions

Investigations on anti-inflammatory actions often focus on the regulation of pro-inflammatory enzymes, such as inducible cyclooxygenase 2 (COX2) [70,71,72,74], inducible nitric oxide synthase (iNOS or nitric oxide synthase, NOS2) [64,71,74,75], or 5-lipoxygenase (5-LO) [72,73], as well as mediators such as chemokines or cytokines. For this purpose, cells were treated with the LCMs and challenged with a pro-inflammatory stimulus or alternatively, isolated enzymes were used. Several LCMs (α-, γ-, δ-13′-COOH; δ-9′-COOH; α-13′-OH) have been tested and reduced the stimulus-induced expression (mRNA or protein) or enzyme activity. In general, 13′-COOH are more potent than the shorter LCMs and the conjugation of LCMs with sulfate abrogates their anti-inflammatory effects [64,70].

Jiang et al. gained first hints on the anti-inflammatory actions of LCMs [70]. A549 cells, which are capable of metabolizing vitamin E, were incubated with TOHs and an inhibition of the arachidonic acid-stimulated COX activity was reported. When the metabolism of vitamin E was suppressed by sesamin, the effects were less pronounced, indicating the involvement of the LCMs as regulatory molecules. For further experiments, the LCMs were extracted from the cell culture medium and their inhibitory capacity on COX activity was tested (half maximal inhibitory concentration (IC_50_): δ-13′-COOH: 4 µM; δ-9′-COOH: 6 µM). The impact of conjugation was tested, and the sulfate LCM conjugates were unable to exert anti-inflammatory effects. In 2016, a comparison of the different types of LCMs was performed, and the LCMs showed similar effects regardless of their origin (isolated from cell culture medium or semisynthetic isolation from *Garcinia kola*) [72]. In RAW264.7 macrophages, the anti-inflammatory action on lipopolysaccharide (LPS)-stimulated COX2 mRNA and protein expression, as well as prostaglandin (PG) release was reported for α-13′-OH [71] and α-13′-COOH [74]. 

The regulation of iNos by the LCMs was studied in RAW264.7 macrophages [64,71,74,75]. The LPS-stimulated iNos mRNA and protein expression as well as release of nitric oxide were reduced by the LCMs tested (α- and δ-13′-OH, α- and δ-13′-COOH) [64]. The inhibitory effect of the LCMs was highly dependent on the structure of the LCMs. The 13′-COOH were more effective than the 13′-OH, while the substitution of the chromanol ring system (α- vs. δ-LCMs) had no influence.

The inhibition of ionophore-induced leukotriene release (leukotriene B_4_) in HL-60 cells and neutrophils was reported with IC_50_ values of 4–7 µM [73]. Furthermore, the activity of isolated 5-LO was inhibited by δ-13′-COOH with IC_50_ values of 0.5–1 µM, which is more effective than the synthetic 5-LO inhibitor zileuton (IC_50_: 3–5 µM) [73]. The inhibition of 5-LO activity by δ-13′-COOH was also confirmed by Jang et al. [72]. An overview of the known anti-inflammatory actions of the different LCMs of vitamin E studied so far is provided in Table 1.

The metabolites of vitamin K have also been shown to exert anti-inflammatory functions. First experiments were carried out with a synthetic 7-carbon carboxylic acid vitamin K metabolite (2-methyl, 3-(2′methyl)-hexanoic acid-1,4-naphthoquinone; K acid 1), which was a more effective inhibitor of LPS-induced IL-6 release from fibroblast than the precursors phylloquinone and menaquinon-4 [80]. In LPS-challenged MG63 osteoblasts the 7-carbon carboxylic acid metabolite as well as the 5-carbon carboxylic acid metabolite (K acid 2) attenuated the expression of IL-6 [81]. Later, the long-chain metabolites of vitamin K (10 to 20-carbon carboxylic acid metabolites) were also synthesized and examined for their anti-inflammatory activity. In LPS-challenged mouse macrophages, these compounds reduced the induction of gene-expression of the inflammatory markers IL-1β, IL-6 and TNFα [82]. However, K acid 1 and K acid 2 were also effective in this study; and it is not possible to estimate, which vitamin K metabolite (either long-chain or short-chain) is the most effective [82]. Interestingly, the minor 7-carbon carboxylic acid metabolite was more effective in MG63 osteoblasts than the 5-carbon carboxylic acid metabolite, and a replacement of the carboxy function by a methyl group made the two metabolites less effective [81]. This is in line with findings for the LCMs of vitamin E. Here, the carboxy metabolite is more effective than the respective TOH precursor with respect to the anti-inflammatory actions (vide supra). However, the in vivo relevance of the regulatory activities of the vitamin K metabolites is a matter of debate, as they increase with vitamin K intake in urine [83], but have not yet been found in human blood or other tissues to the best of our knowledge.

### 4.2. Cancerogenesis and Chemoprevention

The metabolites of vitamin E were investigated with respect to putative anti-cancerogenic, i.e., anti-proliferative and pro-apoptotic, properties in several studies. First experiments revealed that the SCMs inhibit cell proliferation in different cell lines [84,85]. Interestingly, the metabolites as well as the precursor molecules showed different efficiencies, depending on the methylation pattern of the chroman ring and also on the cell type tested [84,85]. Based on the anti-proliferative effects of the SCMs, the interest in the effects of the LCMs aroused. Hence, Birringer et al. investigated the effects of the LCMs α-13′-COOH and δ-13′-COOH as well as α-13′-OH and δ-13′-OH on the proliferation of the human hepatocyte carcinoma cell line HepG2 [77]. Interestingly, both 13′-COOH metabolites effectively caused cell growth arrest, but the hydroxy metabolites did not exhibit anti-proliferative effects. Thus, the introduction of the carboxy group during TOH metabolism renders the molecule active with respect to cell growth arrest. This is supported by the finding that the metabolic precursors, i.e., TOHs, did not affect proliferation of HepG2 cells [77]. As mentioned above, the methylation of the chroman ring alters the efficiency of the molecules. With an effective concentration of 6.5 µM in HepG2 cells regarding the effects on cell growth, the δ-metabolite is more effective than its α-counterpart with 13.5 µM [77]. At first glance, contradictory results were reported for human prostate cancer cells. Here, not only δ-13′-COOH inhibited cell proliferation, but also the hydroxy metabolite α-13′-OH. The LCMs as well as the tested SCMs α-CEHC and γ-CEHC inhibited the proliferation by about 60% in a concentration of 10 µM [76]. Hence, the efficiency of the hydroxy metabolite is likely dependent on the cell type. It is possible that the differences in TOH metabolism in different cell types lead to divergent effects. Interestingly, even differences between different cancer and non-cancer cell lines have been described. The proliferation of the colon cancer cell lines HCT-116 and HT-29 was inhibited by δ-13′-COOH, with IC_50_ values of 8.9 µM and 8.6 µM, respectively [72]. While 10 µM of the LCMs reduce the viability of the cancer cells by around 60%, normal colon epithelial cells showed a reduction of 10–20% at this concentration. Comparable effects were found for the δ-T3 LCM δ-T3-13′-COOH (δ-garcinoic acid), which reduced the viability of the colon cancer cells by about 75%, but the viability of normal colon cells merely by 10–20% [72]. 

The actions of the vitamin E metabolites are comparable to that of the metabolites of vitamin D and vitamin A. The active vitamin D metabolite 1,25(OH)_2_D_3_ has been shown to modulate differentiation and proliferation of colon cancer cells and prostate cancer cells [86]. However, 1,25(OH)_2_D_3_ led to an arrest of most cells that express a functional vitamin D receptor in G0/G1 phase [87]. The actions are mediated by interference with several regulatory proteins, such as epidermal growth factor receptor (EGFR), insulin-like growth factors (IGFs), p21, p27 as well as cyclins and cyclin-dependent kinases (CDKs) [87]. The retinoids are also known for their modulation of the cell cycle. In several cancer cell lines, retinoic acid (RA) led to a cell cycle arrest in the G0/G1 phase via direct or indirect modulation of cyclins, CDKs and cell-cycle inhibitors [88]. Interestingly, TOHs and TOH SCMs have also been linked to cyclins and CDKs. In the human prostate cancer cell line PC3, γ-TOH as well as γ-CEHC led to a strong decrease in cyclin D1 protein expression. In line with this observation, CDK4 and p27 expression are reduced, albeit less pronounced [85]. Moreover, α-TOH and α-CEHC are ineffective with respect to anti-proliferative actions as well as suppression of cyclin D1 and CDK4 [85]. However, to date, no data is available on the action of the vitamin E LCMs on cell cycle regulators, although strong anti-proliferative effects have been shown for this class of metabolites. 

More detailed investigations were carried out on the pro-apoptotic effects of the vitamin E LCMs. Birringer et al. found a significant induction of apoptosis in HepG2 cells treated with 20 µM of α-13′-COOH, δ-13′-COOH or δ-13′-OH [77]. The LCMs induced the cleavage of caspases 3, 7 and 9, and in line with this, the cleavage of the downstream mediator poly-ADP ribose polymerase-1 (PARP-1). Again, the 13′-COOH were more effective in caspase-cleavage and apoptosis induction than the hydroxy metabolite [77]. Moreover, induction of mitochondrial apoptosis by the LCMs was identified as the process leading to apoptosis. This process is accompanied by the formation of reactive oxygen species (ROS). Birringer et al. observed a significant increase in ROS production in cells treated with α- and δ-13′-COOH but not with the hydroxy metabolites and the TOHs [77]. The augmented ROS production was not only measured intracellularly but also intramitochondrial, hence providing evidence for mitochondrial-derived apoptosis. Alterations in the mitochondrial membrane potential supported this finding. Treatment with 20 µM of the LCMs led to a significant reduction of the mitochondrial membrane potential. Interestingly, in this particular case, the α-metabolite was more potent than the δ-metabolites with 60% reduction vs. 20% reduction [77]. The pro-apoptotic actions of the δ-LCMs of vitamin E were confirmed in colon cancer cells [72]. Early and late apoptosis were induced by δ-13′-COOH and δ-T3-13′-COOH. The activation of caspase-9 and cleavage of PARP found by Birringer et al. [77] were confirmed in colon cancer cells [72]. Moreover, an induction of the autophagy marker microtubule-associated protein 1A/1B-light chain 3 (LC3)-II was found. Jang et al. assumed that alterations in sphingolipid metabolism caused by the carboxy-LCMs are the reason for the induction of apoptosis. Indeed, both δ-13′-COOH and δ-T3-13′-COOH increased total ceramides, dihydroceramides and dihydrosphingosines, while all measured sphingomyelins were decreased. Inhibition of sphingosine biosynthesis revealed that LC3-II expression but not PARP-cleavage is modulated by the LCMs via alterations in sphingolipid metabolism [72]. 

Taken together, there are several similarities between the metabolites of vitamins A, D and E with respect to anti-cancerogenic properties. Data on anti-cancerogenic effects of vitamin K metabolites, however, are sparse. Merely synthetic carboxylic derivatives of menaquinone with different side-chain lengths have been studied [89]. The biologically most abundant 5-carbon carboxylic acid metabolite (K acid 2) was not included in this study and the 7-carbon carboxylic acid metabolite (K acid 1) was the structure with the shortest side-chain. Interestingly, the growth-suppressing effect on hepatocellular carcinoma cells increased with the length of the side chain of the carboxy derivatives, except for the full-length metabolite, which was as effective as the 7-carbon carboxylic acid metabolite. Conversely, menaquinone itself was completely ineffective, showing nicely that the introduction of a carboxy function activates the compound. Blocking of the effects with chemical antagonists suggested that the derivatives act through caspase/transglutaminase-related signaling [89]. The above mentioned disruption of mitochondrial function by the LCMs of vitamin E has also been described for the metabolites of vitamin A [90], and induction of apoptosis by 1,25(OH)_2_D_3_ via mitochondrial pathways (e.g., via B-cell lymphoma (BCL)-2 and BCL-xL) in breast, colon and prostate cancer cells are also known [87]. Based on their anti-proliferative and pro-differentiation actions but also due to the induction of cell death, retinoids are used for treating certain types of cancer [91]. Vitamin A metabolites were successfully used in the treatment of acute promyelocytic leukemia (ATRA and 13-*cis*-RA, 13*c*RA), squamous cell skin cancer and neuroblastoma (13*c*RA), lung cancer (ATRA) and Kaposi’s sarcoma (9-*cis*-RA, 9*c*RA). Beneficial effects of retinoids in cancer prevention have also been observed. These properties can be explained by the targeting of regulators of cell cycle progression by retinoids. The expression of the CDK inhibitors p21 and p27 is regulated by ATRA via RARβ2 upregulation, and retinoic acid has been shown to stimulate the degradation of cyclin D1, leading to a suppression of CDK activity [91]. Interestingly, TOHs as well as SCMs of vitamin E modulate cyclins, CDKs and CDK inhibitors [85]. Albeit the LCMs of vitamin E efficiently suppress proliferation, the identification of effects on regulators of cell cycle progression is pending. However, given that ‘decreased proliferation is one of the best biomarkers of a cancer preventive effect’ [91], vitamin E and its metabolites are promising compounds for cancer prevention.

### 4.3. Cellular Lipid Homeostasis

To date, the effects of the LCMs of vitamin E on cellular lipid homeostasis have not been investigated extensively. However, the regulation of key metabolic pathways in foam cell development of macrophages by the LCMs were of particular interest in a study by Wallert et al. [63]. Here, the regulation of the expression of the cluster of differentiation 36 (CD36), the uptake of oxidized low density lipoprotein (oxLDL), phagocytosis and the intracellular storage of lipids were investigated [63]. For this, the monocytic THP-1 cell line, which can be differentiated to macrophage-like cells, was used. In differentiated macrophages, the LCMs α-13′-OH and α-13′-COOH induced the expression of CD36 mRNA and consequently CD36 protein levels. In contrast, the precursor α-TOH exerted opposite effects on CD36 mRNA and protein. Whereas α-TOH reduced the expression of CD36 at a concentration of 100 µM, the α-LCMs induced the expression of CD36 in concentrations of 5 and 10 µM, respectively [63]. Thus, the α-LCMs not only act in a different way than their precursors, but appeared to be also significantly more potent. Interestingly, similar effects were described for the lipid soluble vitamin A. Langmann et al. found that the precursor β-carotene is less effective in inducing expression of CD36 than its metabolites ATRA and 9*c*RA in human monocytes and macrophages [92]. The authors stated that the metabolites 9*c*RA and ATRA displayed high biological activity [92], while the precursors retinol and β-carotene were only marginally metabolized, an observation that parallels the characteristics of the LCMs of vitamin E with respect to their reported serum concentrations [63,93]. The effects of vitamin A metabolites are better characterized than that of the LCMs of vitamin E. It was repeatedly shown that the metabolites of vitamin A regulate CD36 expression in macrophage cell models. The metabolite 9*c*RA induced CD36 mRNA [94,95] and protein expression [95] in human THP-1 macrophages. ATRA increases expression of CD36 mRNA in THP-1 cells [96] and CD36 protein in THP-1 and HL60 macrophages [96,97]. The induction of CD36 expression by ATRA and 9*c*RA has been confirmed in primary human monocytes and macrophages [92,96] to show the physiological relevance in non-cancer cells. With the same intention, it was also shown that the LCMs of vitamin E induced CD36 expression in peripheral blood mononuclear cell (PBMC)-derived primary human macrophages [63].

The scavenger receptor CD36 mediates the uptake of the modified lipoprotein oxLDL [98], a process that in turn stimulates CD36 expression [99]. Given the induction of the expression of CD36 by the LCMs of vitamin E under basal conditions (vide supra), a further stimulation by oxLDL treatment could be expected. As the uptake of oxLDL is a hallmark of macrophage foam cell formation, Wallert et al. examined whether preincubation of THP-1 macrophages with the LCMs of vitamin E affects the oxLDL-induced expression of CD36 [63]. As expected, CD36 expression was induced by oxLDL treatment. Pre-treatment with α-TOH suppressed the induction by oxLDL. In contrast, the pre-incubation with the LCMs augmented the induction of CD36 expression by oxLDL. These findings resemble the reaction of the cells in the absence of oxLDL to α-TOH and its LCMs. Given the higher CD36 expression in the presence of the LCMs, the uptake of oxLDL should in turn be induced in LCM-treated macrophages. However, pre-incubation of the macrophages with the LCMs for 24 h led to decreased oxLDL uptake. Incubation with both, α-13′-OH or α-13′-COOH, decreased the uptake by about 20%. This effect was again confirmed in PBMC-derived macrophages. Here, oxLDL uptake was decreased by α-13′-OH pre-treatment by 24% and by α-13′-COOH pre-treatment by 20%, respectively [63]. The LCMs of vitamin E thus exerted unexpected effects on oxLDL uptake. As mentioned before, vitamin A metabolites also caused increased CD36 expression, but the metabolite 9*c*RA induced the binding and uptake of oxLDL in THP-1 macrophages as expected [94]. Generally, an activation of RXR leads to an augmented association of oxLDL to THP-1 macrophages [100]. However, 9*c*RA also promoted the degradation of oxLDL and the cholesterol efflux via ATP binding cassette transporters, thus leading to a net depletion of cholesterol esters. Triglyceride levels were apparently not affected, neither by oxLDL treatment nor combination with 9*c*RA [94]. In contrast, in the study of Wallert et al. on the LCMs of vitamin E, oxLDL treatment of the macrophages led to an increase of neutral lipids in the cells. Preincubation with the LCMs diminished the oxLDL-induced neutral lipid accumulation [63]. However, the contradictory results on the effects of the LCMs on CD36 expression and oxLDL uptake required an alternative explanation how the LCMs decrease oxLDL uptake. Thus, Wallert et al. focused on phagocytosis as an alternative uptake mechanism for oxLDL [101]. Indeed, treatment of the macrophages with α-13′-OH led to an inhibition of phagocytotic activity of 16% and with α-13′-COOH of 41%, respectively [63]. Hence, the inhibition of phagocytosis by the LCMs might explain the discrepancy between their effects on CD36 expression and oxLDL uptake in this study.

Taken together, the metabolites of vitamin E and vitamin A induce the expression of CD36 in macrophages. However, their effects on oxLDL uptake are different. While the vitamin A metabolite 9*c*RA induces oxLDL uptake, the LCMs of vitamin E reduce it. In contrast to vitamin A and vitamin E metabolites, the metabolite of vitamin D, 1,25(OH)_2_D_3_ has been shown to reduce the expression of CD36 mRNA and protein in oxLDL-treated macrophages obtained from diabetic subjects. Concomitantly, oxLDL and cholesterol uptake are decreased [102,103]. Hence, the vitamin D metabolite as well as the vitamin E LCMs suppress macrophage foam cell formation and may thus exert positive effects in the context of atherosclerosis prevention.

### 4.4. Interaction with Pharmaceuticals

The interaction of the vitamin E LCMs with pharmaceuticals was tested by analyzing the regulation of P-glycoprotein (P-gp). P-gp regulates, inter alia, the intracellular concentration of pharmaceuticals and its expression is regulated by various transcription factors, including heat shock transcription factor 1, nuclear factor Y and the pregnane X receptor (PXR) [104,105].

Several vitamin E forms and their metabolites (α-TOH, α-T3, α-13′-COOH, α-CEHC, γ-TOH, γ-T3, γ-CEHC and plastochromanol-8) were used and the regulation of P-gp expression was analyzed in human epithelial-like colon LS180 cells [78]. Only α-13′-COOH and γ-T3 induced P-gp expression and α-T3, α-13′-COOH as well as γ-T3 induced the activity of PXR in a reporter gene assay. In case of vitamin E supplementation, an interaction with the metabolic handling of pharmaceuticals might be possible.

### 4.5. Regulation of LCM Formation

The regulatory processes, which modulate the metabolism of vitamin E, are largely unknown. In this context, two key issues are important: (i) Apart from CYP4F2 and CYP3A4, the full set of enzymes involved in the first steps of the catabolism of vitamin E remains to be identified, and (ii) the mechanisms by which vitamin E metabolism is regulated have not yet been sufficiently unraveled. However, the upregulation of CYP4F2 protein expression by α-13′-OH in human HepG2 liver cells was reported recently [79], pointing to a positive regulatory feedback loop. If this concept holds true, the enhancement of metabolism by products would be a new facet for the fat-soluble vitamins, as the metabolism of vitamin A and D is mainly regulated negatively by their metabolic products [54,106].

The aldehyde- and alcohol-dehydrogenases have been suggested to be responsible for the ω-oxidation steps and the enzymes for branched-chain fatty acids might catalyze the subsequent β-oxidation [107]. Following the identification of the specific set of enzymes required for vitamin E metabolism, a major aim will be the characterization of the regulatory factors, which modulate the metabolism of vitamin E.

## 5. Structure-Specific Effects

To get deeper insights into the specificity of the regulatory effects of the LCMs of vitamin E, a structure-activity study was conducted [64]. For this purpose, substances were used that represent specific substructures of the LCMs or their precursors. The chromanol ring system was mirrored by the SCM α-CEHC and the modified side-chain was represented by the branched-chain fatty acid pristanic acid. Furthermore, the α- and δ-forms of 13′-OH and 13′-COOH were used to study the influence of the side-chain modification. Overall, the application of α- and δ-forms of LCMs and their precursors (α-TOH, α-13′-OH, α-13′-COOH, δ-TOH, δ-13′-OH, δ-13′-COOH) should clarify the importance of the substitution of the ring-system. The regulation of CD36 and iNos by the test compounds was similar for all of the LCMs, but neither the precursors nor their substructures were able to cause the same effects on the expression of the target genes as the LCMs. The substitution of the chromanol ring system had no influence (α- and δ-forms), while the modification of the side-chain (oxidation of TOH to 13′-OH and 13′-COOH) was highly relevant for the effects. Overall, the 13′-COOH was most potent in this study. Based on these specific regulations the existence of specific regulatory molecular pathways for the LCMs has been suggested. 

## 6. Receptors of Vitamin Metabolites

As indicated above, the lipid-soluble vitamins A and D need a conversion to their active metabolites to exert their effects. These metabolites are either bound intracellularly and transferred to the receptor or directly bind the receptor. The receptors for the vitamin A metabolites, RARs and RXRs, were identified in the late 1980s [108,109,110,111]. Evidence for binding proteins for the active vitamin D metabolite 1,25(OH)_2_D_3_ was already provided in the 1970’s [112,113]; however, cloning of the human vitamin D receptor also succeeded in the late 1980’s [114]. In contrast, no specific receptor for vitamin E and/or its metabolites has been identified yet. Interestingly, the metabolites of vitamin A and D act through nuclear receptors. This class of transcription factors can roughly be divided into more specific and rather unspecific members. The vitamin D receptor can be categorized as a more specific receptor, as it is activated by its endogenous ligand 1,25(OH)_2_D_3_ already at sub-nanomolar concentrations [115,116]. This feature is also shared by steroid hormone receptors (estrogen receptor, androgen receptor, ergosterone receptor, cortisol receptor), the thyroid hormone receptor and RARs. The RARs specifically bind ATRA, and also 9*c*RA with lower affinity [117]. The specificity of the nuclear receptors is mainly determined by the structure of the ligand binding pocket. Specific receptors have a relatively small ligand binding pocket, which allows only a limited number of molecules to interact. In contrast, the so-called adopted orphan receptors have a larger ligand binding pocket, allowing the activation of the receptor by a larger number of ligands [115]. Members of this group are the liver X receptors (LXRs), farnesoid X receptor (FXR), peroxisome proliferator-activated receptors (PPARs) and RXRs. The latter have been shown to bind the vitamin A metabolite 9*c*RA [118]. However, it is not entirely accepted that 9*c*RA represents the endogenous ligand for RXR [119]. Nonetheless, the example of 9*c*RA opens the possibility that vitamin metabolites act through highly specific receptors but also through rather unspecific ones. 

Following the concept that the LCMs of vitamin E represent biologically active metabolites similar to 1,25(OH)_2_D_3_, ATRA and 9*c*RA, these molecules might also exert their effects through nuclear receptors. Indeed, Podszun et al. reported an activation of PXR by α-13′-COOH in the human colon adenocarcinoma cell line LS180 [78] (for detailed information, the reader is referred to the section ‘Interaction with pharmaceuticals’). Interestingly, α-T3 and γ-T3 were also able to activate PXR, while α-TOH and γ-TOH as well as the SCMs α-CEHC and γ-CEHC failed to activate PXR [78]. These findings confirm earlier findings in HepG2 cells only in part. In HepG2 cells transfected with PXR and a CAT (chloramphenicol acetyltransferase) reporter gene, α-T3 and γ-T3 efficiently activated PXR-mediated gene transcription, but α-TOH, γ-TOH and δ-TOH were also able to induce the expression of the reporter gene via PXR [120]. In contrast, the SCMs α-CEHC and α-CMBHC were not able to activate PXR in this study and the LCMs were not tested [120]. Taken together, the T3s reliably activate PXR but the effects of the TOHs need further investigation. Possibly, LS180 and HepG2 metabolize TOH with different efficiency, in turn determining the amounts of LCMs formed as PXR-activating metabolites. Hence, the observed effects of TOHs in HepG2 might be explained by the intracellular formation of the LCMs. However, further investigations on the cell-type specific metabolism of TOH are needed to confirm this hypothesis. Further, with PXR a rather unspecific nuclear receptor is identified for TOHs and their LCMs. As a general sensor for toxic compounds and xenobiotics, PXR has a large ligand binding cavity, which allows the binding of a wide range of ligands [121]. Thus, it is not surprising that PXR has been described as a receptor of vitamin K [122,123], and it has been reported that several menaquinone derivatives activate PXR [124]. Unfortunately, the biologically occurring carboxy derivatives were not included in this study. Hence, merely speculations about the activity based on structure-function-relationships are possible. A reporter gene assay revealed that a terminal phenyl group enhances the activity of the derivatives, while a terminal hydroxy group diminished it compared to the unmodified menaquinone [124]. In conclusion, a more hydrophobic side chain leads to an increased activity on PXR. Hence, the natural metabolic products in humans bearing a terminal carboxy group are likely less potent with respect to the activation of PXR. However, this concept is in contrast to the findings for vitamin E. The TOH precursors are unable to activate PXR, while the LCM α-13′-COOH activates it [78]. Hence, further studies are needed to clarify whether vitamin K metabolites are physiological ligands for the rather unspecific nuclear receptor PXR, like their metabolic precursor menaquinone and the LCMs of vitamin E.

Given that RXR as a receptor for the vitamin A metabolite 9*c*RA is also rather unspecific, it might be possible that the LCMs of vitamin E also act through PXR. However, it is questionable whether all of the reported biological effects of the LCMs, i.e., anti-inflammatory actions, anti-cancerogenic features, and effects on cellular lipid homeostasis (please refer to the respective sections here) can be ascribed to PXR activation. Hence, further investigations aiming at the identification and characterization of receptors for the LCMs of vitamin E LCMs are highly required. Strategies for the identification of further receptors or a receptor specific for the LCMs of vitamin E might be the use of target fishing approaches, gene expression arrays, knockdown/knockout studies, as well as reporter gene assays and ligand binding studies.

## 7. Conclusions

With the detection of the LCMs of vitamin E in human serum, an important hint for the possible action of these metabolites as signaling molecules was provided. Several studies reinforced this hypothesis by the characterization of the biological effects of the LCMs, as summarized in Figure 2. Interestingly, the LCMs act more potent and in part even contrary to their metabolic precursors. Some of the controversial effects reported for vitamin E might be therefore explained by the action of the LCMs. The evidence of circulating α-LCM in human blood (nanomolar concentrations) provides a new perspective in vitamin E research [63]. Therefore, the LCMs must be seriously considered to correctly interpret the effects of vitamin E in humans, beside the better studied TOHs and T3s. So far, only a few studies have focused on this class of compounds. However, based on our current knowledge and our studies in progress, we speculate that the LCMs comprise a new class of regulatory molecules. These molecules can exert effects that are different from their metabolic precursors, complicating the interpretation of studies on the effects of vitamin E in vivo. Nevertheless, the LCMs share properties with their precursors but also exert unique or even adverse effects. It is evident that the LCMs and their precursors act in the same manner with respect to the modulation of COX2 and 5-LOX activity, but it is of note that the LCMs are significantly more potent than their precursors. Furthermore, the LCM can act in areas where the TOHs are virtually not effective. A prime example is the regulation of COX2 expression. Hence, the LCMs may indeed play a role in mediating some of the effects of vitamin E in the human body although blood concentrations are significantly lower than those of TOH. So far, blood concentrations are the only valid value for the systemic distribution of the LCMs of vitamin E in the human body. However, based on preliminary data of unpublished in vitro and in vivo studies of our group, we can hypothesize that the LCMs of vitamin E may also accumulate in different parts of the human body, where they reach concentrations higher than in blood. Further studies are required to study this issue in more detail and to differentiate between physiologic (at low concentrations) and pharmacologic (at high concentrations) actions of the LCMs.

To sum up, the LCMs could be regarded as the metabolically activated forms of vitamin E. This is in line with the metabolic activation of the other lipid-soluble vitamins A and D. Consequently, the concept of metabolic activation established for vitamin A and D could now be extended to vitamin E. Thus, a general concept for the biological activity and modes of action of the lipid-soluble vitamins could be defined.

## Figures and Tables

**Table 1:** Overview of anti-inflammatory actions of the LCMs of vitamin E.

Targets	Cells	Effects	Substances	Refs.
COX2	A549 cells	Reduced activity in arachidonic acid-pre-induced cells	γ-13′-COOH	[70]
			δ-13′-COOH	[70,72]
			δ-9′-COOH	[70]
	Isolated enzyme	Inhibition of activity	δ-13′-COOH	[70]
			δ-9′-COOH	
	RAW264.7	Inhibition of LPS-stimulated mRNA and protein expression, as well as reduced PG release	α-13′-OH	[71]
			α-13′-COOH	[74]
iNos	RAW264.7	Inhibition of LPS-stimulated mRNA and protein expression, as well as reduced release of nitric oxide	α-13′-OH	[64,71,74,75]
			α-13′-COOH	
			δ-13′-OH	
			δ-13′-COOH	
5-LO	Isolated enzyme	Inhibition of activity	δ-13′-COOH	[72,73]
	HL-60 neutrophils	Reduced activity and LT release in pre-induced cells	δ-13′-COOH	[73]

PG, prostaglandin; LT, leukotriene.
